# Interventions to Address Financial Vulnerability in Dementia: A Scoping Review

**DOI:** 10.1093/geroni/igaf122.4148

**Published:** 2025-12-31

**Authors:** Shadi Gholizadeh, Megan Harris, Lucas Melville

**Affiliations:** The Key, Coto de Caza, California, United States; TheKey, San Diego, California, United States; TheKey, San Diego, California, United States

## Abstract

Older adults living with dementia and other cognitive changes are at elevated risk for financial exploitation, mismanagement of funds, and financial abuse. Despite this, relatively few interventions have been developed to address financial vulnerability in home and community settings, especially when care is also being provided. This scoping review aimed to identify and summarize peer-reviewed studies describing interventions targeting financial harm among people with dementia. PubMed and PsycINFO were searched using terms related to cognitive impairment, financial exploitation, and intervention. Of 39 articles retrieved, 7 were reviewed in full, and 5 met inclusion criteria. Interventions included: a home photo-assessment tool to identify environmental triggers for delusions of theft; a telehealth dementia care coordination model using unlicensed navigators to address financial and legal concerns; a policy-focused interview study with safeguarding professionals on risks tied to personal budget misuse; a multidisciplinary forensic center model that increased referrals for conservatorship; and a behavioral-technology study identifying commonly lost objects to inform assistive robotics. Across studies, interventions varied in scope and rigor. Most were small-scale, lacked formal evaluation, and none were randomized controlled trials. However, they highlighted promising strategies to reduce financial harm through environmental, relational, technological, and legal approaches. Most targeted early to moderate dementia and were caregiver-inclusive. This review underscores the urgent need for scalable, rigorously evaluated interventions that promote financial safety as part of dementia care planning, while preserving autonomy, reducing caregiver strain, and enabling interdisciplinary collaboration across care, legal, and community settings.

